# Using the State Plan Index to Evaluate the Quality of State Plans to Prevent Obesity and Other Chronic Diseases

**Published:** 2005-03-15

**Authors:** Diane O Dunět, Frances D Butterfoss, Robin Hamre, Sarah Kuester

**Affiliations:** Health Scientist, Centers for Disease Control and Prevention (CDC), National Center for Chronic Disease Prevention and Health Promotion, Division of Nutrition and Physical Activity, Chronic Disease Nutrition Branch; Health Promotion & Disease Prevention, Center for Pediatric Research, Eastern Virginia Medical School, Norfolk, Va; Nutrition and Physical Activity Program to Prevent Obesity and Other Chronic Diseases, Centers for Disease Control and Prevention (CDC), National Center for Chronic Disease Prevention and Health Promotion, Division of Nutrition and Physical Activity, Atlanta, Ga; Nutrition and Physical Activity Program to Prevent Obesity and Other Chronic Diseases, Centers for Disease Control and Prevention (CDC), National Center for Chronic Disease Prevention and Health Promotion, Division of Nutrition and Physical Activity, Atlanta, Ga

## Abstract

**Introduction:**

Implicit in public health planning models is the assumption that good public health plans lead to good programs, and good programs lead to desired health outcomes. Despite considerable resources that are devoted to developing plans, public health agencies and organizations have lacked a tool for evaluating the finished product of their planning efforts — the written plan itself — as an important indicator of progress. To address the need for an instrument to assess the quality of state plans designed to prevent and control chronic diseases, we created and tested the State Plan Index and used it to evaluate the quality of nine state plans aimed at preventing and reducing obesity.

**Methods:**

The State Plan Index was developed under the auspices of the Centers for Disease Control and Prevention (CDC) in collaboration with public health experts in federal, state, and academic settings. The State Plan Index included 55 items related to plan quality arranged into nine components. Each item was rated on a Likert scale from 0 to 5, with 5 being the highest rating. Each plan also received a separate overall plan quality score using the same scale. Each state plan was evaluated by four or five raters using the State Plan Index. For each plan, the 55 items were averaged to calculate an item average score, and a subscore was calculated for each State Plan Index component. Finally, five states also self-rated their own plans (self score).

**Results:**

The mean item average score for all plans was 2.4 out of 5.0. The range of item average scores was 1.0 to 3.0. The component of the State Plan Index with the highest mean component score (3.3) was Presentation of Epidemiologic Data on Disease Burden. The components with the lowest component scores were Resources for Plan Implementation (0.7); Integration of Obesity Efforts with Other Chronic Disease Efforts (1.7); and Program Evaluation (2.0). Plan quality was rated higher when based on the single overall plan quality score assigned by raters. In addition, self scores were consistently and substantially higher than rater-assigned scores.

**Conclusion:**

Evaluation of plans early in the life of programs can be used to strengthen existing programs and to guide programs newly engaged in chronic disease prevention planning. The CDC has used the State Plan Index evaluation results to guide technical assistance, plan training sessions, and enhance communication with state staff about plan content, quality, and public health approach. Some state program directors self-evaluated their obesity draft plan and used the evaluation results to strengthen their planning process and to guide plan revisions. Other states have adapted the State Plan Index as a framework for new planning efforts to prevent obesity as well as other chronic diseases.

## Introduction

Public health experts promote planning at the state and community levels in order to achieve desired public health outcomes (1). With the support of the Centers for Disease Control and Prevention (CDC), substantial public health resources have been devoted to state planning in areas such as bioterrorism preparedness, school health, and tobacco control (2). Drawing on long traditions of planning and organizational science, public health and policy experts have developed an array of planning models (3-7) as well as tools with which to assess infrastructure (8-10), design interventions (11-13), and manage ongoing data collection (14). The availability of different planning models provides public health practitioners with the flexibility to not only match an appropriate model with an intended goal but also to use a model (or a combination of model elements) that is compatible with the norms, expectations, and acceptability of organizations and community stakeholders (15). Implicit in planning models is the assumption that good plans lead to good programs, and good programs lead to desired health outcomes. Thus, the quality of a plan deserves focused attention from evaluators and public health practitioners.

Although many planning models include evaluation, this evaluation is often in the context of assessing the effectiveness of strategies selected through the planning process or tracking the status of the plan's implementation. Recent attention has turned to the plans themselves — the finished products of the planning process — as indicators of progress. However, plan evaluations have been limited to inventories and descriptions of the content of state plans that address various chronic diseases; they have not directly addressed plan quality. (See, for example, Abed et al [16].) More typically, evaluation of state plans is informal, as when program staff judge a plan primarily on the basis of their own expertise. In the current study, comprehensive state obesity prevention plans were systematically evaluated for quality. The plans were developed by states that receive funding and technical assistance under CDC cooperative agreements for obesity through the CDC's Obesity Prevention Program described below.

### The CDC Obesity Prevention Program

Obesity in the United States has reached epidemic proportions. Among adults in the United States, the prevalence of overweight is approximately 65.7%, and the prevalence of obesity is approximately 30.6% (17). Body mass index (BMI) is calculated as a person's body weight in kilograms divided by the height squared in meters. Adults with a BMI of 25 to 29.9 are considered overweight, while adults with a BMI of ≥30 are considered obese (18).

In children, weight status is determined using sex-specific growth charts for BMI-for-age, with overweight status defined as a BMI at or greater than the 95th percentile (19). Since 1980, the prevalence of overweight has doubled for children aged two to 11 years, while in adolescents aged 12 to 19 years, the prevalence of overweight has more than tripled (17). *The Surgeon General's Call to Action to Prevent and Decrease Overweight and Obesity* (20), published in 2001, states that the cost in the United States of overweight and obesity and their complications is estimated at $117 billion annually. Obesity in this country is both epidemic and costly.

The factors that contribute to obesity are many and varied, as are the public health strategies needed to address obesity. In 2000, the CDC launched its state-based Nutrition and Physical Activity Program to Prevent Obesity and Other Chronic Diseases (CDC Obesity Prevention Program). The CDC Obesity Prevention Program maintains a Web site with detailed information about the program and links to resources available from www.cdc.gov/nccdphp/dnpa/obesity/state_programs.

### State plans for obesity prevention

The CDC Obesity Prevention Program is based on a familiar public health model: state health departments are funded by a federal agency to develop comprehensive plans and are provided with ongoing technical assistance, training, and other resources. In 2000, the CDC Obesity Prevention Program awarded six state health departments (California, Connecticut, Massachusetts, North Carolina, Rhode Island, and Texas) an average of $350,000 per year through cooperative agreements. In 2001, an additional six states (Colorado, Florida, Michigan, Montana, Pennsylvania, and Washington) received similar awards. As of July 2004, 23 state health departments were funded for obesity program planning and capacity building, and five were funded by the CDC to implement their state plan for obesity.

The CDC promotes a three-pronged approach to obesity planning: behavior change, environmental change, and policy change. The cooperative agreements between the CDC and states have the following goals: promote development and implementation of community nutrition and physical activity plans for obesity prevention and control; decrease levels of obesity or reduce the rates of growth of obesity in communities reached through interventions; increase physical activity and improve dietary behaviors in communities reached through interventions; and increase interventions, policies, environmental supports, and/or legislative actions for improved nutrition and physical activity.

In developing obesity plans, states are encouraged to draw upon local resources, develop community support, and identify political, economic, and environmental factors that may act as barriers or facilitators to change. Thus, flexibility is important in planning models, and approaches should be compatible with the needs of a state and its current circumstances. At the same time, however, sound principles of public health practice and evidence-based strategies must be used to benefit from theory, scientific research, and program evaluation. Because obesity is a factor in many other chronic diseases, such as diabetes, cardiovascular disease, cancer, and arthritis, states are strongly encouraged to find ways to integrate strategies for obesity control into existing program structures. Although this approach adds complexity because of the many and varied stakeholder interests involved, integrated strategies can facilitate unified public health messages and offer potential cost savings through shared resources (21).

Every state funded by the CDC Obesity Prevention Program is required to develop and implement a state plan. Assessing plan quality serves as an important early indicator of whether states are on track. In addition, the quality of state plans is one of several indicators used by the CDC Obesity Prevention Program to self-monitor its effectiveness in providing guidance and technical assistance for planning.

Because no satisfactory evaluation instrument was available to assess state plan quality, a new instrument was developed for this purpose called the State Plan Index (SPI). As described in detail in "State Plan Index: A Tool for Assessing the Quality of State Public Health Plans" in this issue of *Preventing Chronic Disease* (22), the SPI was developed by a team at the CDC led by Butterfoss and Dun?t in collaboration with more than 100 public health experts in federal, state, and academic settings. The objective for this evaluation was to assess the quality of state plans developed by states that receive funding under the CDC cooperative agreements described above.

## Methods

The SPI used for this evaluation consisted of 55 items arranged into nine components:

1)  Involvement of Stakeholders; 2) Presentation of Data on Disease Burden and Existing Efforts to Control Obesity; 3) Goals; 4) Objectives; 5) Selecting Population(s) and Strategies for Intervention; 6) Integration of Strategies with Other Programs and Implementation of Plan; 7) Resources for Implementation of Plan; 8) Evaluation; and 9) Accessibility of Plan. A six-point Likert scale was used to score each item, each component, and the overall quality of a plan. "Not Addressed" was scored as 0. Consistent with the findings of the formative evaluation on weighting conducted during SPI development (22), SPI items were weighted equally, as were the nine components.

Nine of the ten state plans used in the pilot test of the SPI (22) were evaluated. The nine states were Colorado, Florida, Massachusetts, Michigan, North Carolina, Oregon, Pennsylvania, Texas, and Washington. As of June 1, 2003, these were the only states in the United States to have completed a full draft or final version of a state plan for obesity. One plan from the pilot test that was not a full draft was not included in the evaluation. With the exception of Oregon's, all of the plans were developed with the support of the CDC's cooperative agreement for obesity. Both the SPI and state plans were developed during the same time frame; therefore, state staff did not have the benefit of the SPI as a tool during their planning process. State staff were invited to voluntarily participate in this evaluation by sharing their plans for review and by serving as raters.

Written state plans were provided directly to the CDC by each state or were downloaded from the state's Web sites. During August and September 2003, 41 SPI ratings were completed on the nine state obesity plans by 18 volunteer raters. The raters were recruited as follows: nine from states funded through the CDC's Obesity Prevention Program; five from nonfunded states recruited through the Association of State and Territorial Public Health Nutrition Directors (ASTPHND); one paid independent public health consultant who rated all nine plans; and three CDC Obesity Prevention Program staff, who rated five to nine plans each.

The intention was to have each plan assessed by five raters consisting of one to two state staff from CDC-funded states; one member of ASTPHND; the independent public health expert consultant; and one CDC Obesity Prevention Program staff member. Because some raters did not complete the ratings within the time allotted, the total number of ratings for this analysis was 41 rather than 45. Raters were assigned plans on the basis of suggestions from the CDC Obesity Prevention Program staff members, who matched state plans with raters they believed were least likely to be familiar with the obesity prevention efforts in that state. This approach was intended to avoid raters' consideration of any background information not included in the written plan. An initial plan to blind reviewers to the names of states on hard copies of plans was determined impracticable, especially because some SPI items involve considering how well plans respond to local conditions. Also, information throughout the plan such as epidemiologic data, the names of partners, or a governor's endorsement made it impossible to conceal state names without the possibility of distorting important details in the plan.

Written instructions were provided to raters on the use of the SPI, and a telephone conference was held for orientation. No formal training session was conducted because the SPI was designed to be used without special training. Raters were given a three-week time period to review and rate plans. No instructions were given on the order in which plans were to be reviewed by each rater. Raters provided scores by marking paper copies of the SPI. For these ratings of state obesity prevention plans, the standard was not a comparison or control group; rather, each state plan was measured against the explicit ideal set forth in the SPI.

In the process of planning this evaluation and developing the SPI, the CDC expanded the scope of the evaluation beyond its initial objective of assessing plans to include a process for providing narrative feedback to states. Raters' narrative comments on individual SPI items and major plan components, as well as overall impressions of plans, were provided to the CDC electronically and compiled by the authors.

After the ratings were completed, telephone debriefings were held to discuss raters' experiences in the evaluation process and their reactions to using the SPI to assess state plans. The evaluation also served as one of the field tests of the SPI. As a result of comments received from raters, the SPI was slightly modified by subdividing five of the 55 SPI items used for this evaluation. The final 60-item version of the SPI is available from the CDC Obesity Prevention Program Web site.

States were encouraged to use the SPI to evaluate their own plan, especially for plans that were not yet finalized and disseminated. Five state program directors or program coordinators conducted self ratings and shared the results with the CDC.

SPSS (SPSS Inc, Chicago, Ill), a statistical software program, was used for analysis of the results. The item average score was calculated for each state; the item average score is the mean of the 55 individual item scores assigned by each rater, averaged across raters for each state. Raters also provided an additional score to represent their judgment of the overall quality of a plan (overall plan quality score). An average overall plan quality score was calculated for each state. The correlation coefficient was calculated to measure the association between scores based on an average of 55 SPI items rated individually and scores based on the single overall plan quality score assigned by the raters.

Because of the small number of states evaluated, the results were not stratified on demographics or other variables. In discussing preliminary results of this evaluation with stakeholders, suggestions were made on variables that might have influenced a state's ability to produce a quality plan. Therefore, correlation analysis was used to explore the potential relationship between the quality of state plans and other variables, including prevalence of adult obesity in the state, state population size, personal income per capita, the length of time the state had received CDC funding for obesity, and the objective review panel score received on the state application for funding under the CDC cooperative agreement.

## Results

### Item average scores and overall plan quality scores

As shown in Table 1, item average scores (average of 55 SPI items) ranged from a high of 3.0 to a low of 1.0 on a scale of 0 to 5. The mean item average score for the nine plans was 2.4, and the median score was 2.6.

State averages of overall plan quality score ranged from a high of 4.3 to a low of 2.3 on a scale of 0 to 5 (Table 1). The mean overall plan quality score was 3.4, and the median score was 3.5. Although overall plan quality scores and the item average scores were highly correlated (Pearson r^2 ^= 0.88, *P* ≤ .01), the raters consistently made an upward adjustment when assigning an overall plan quality score.

Self-rating scores were consistently — and substantially — higher than the scores assigned by the other raters. As shown in Table 1, the mean of rater-assigned scores for these five states was 2.6, whereas the mean for self ratings was 4.7, or almost double (median score = 5.0).

### Component scores

Table 2 shows state scores organized by SPI component. A component score is the average of rater-assigned scores for all of the items within that component by all raters of that plan. Mean scores for the SPI components ranged from a high of 3.3 for Presentation of Data on Disease Burden and Existing Efforts to Control Obesity to a low of 0.7 for Resources for Implementation of Plan. Scores in the Resources component ranged from 0 to 1.6, with even the highest scores falling well below ideal. Examples of SPI items in this component included whether or not the lead agency for the plan was identified, how resources would be provided to local partners, and whether the plan addressed issues related to sustainability of efforts.

The component Integration of Strategies with Other Programs and Implementation of Plan was the only other SPI component where the highest score achieved by a state plan was less than 3.0. The mean score for this component was 1.7, with state component scores ranging from a low of 0.7 to a high of 2.6. Examples of SPI items in this component included how strategies will be integrated with existing programs that focus on chronic diseases, prevention, education, and service delivery; and how existing or potential partners (government, community-based, faith-based, business/industry, and private organizations) will be involved to implement the plan. The SPI component with the greatest variability in the range of scores was Presentation of Data on Disease Burden and Existing Efforts to Control Obesity; one state scored 0.0 because no epidemiologic data were presented in the plan, and another state scored 4.5.

At least one state plan scored 4.0 or higher in at least one of the following components: Involvement of Stakeholders (one state), Presentation of Data on Disease Burden (two states), Objectives (one state), and Accessibility of Plan (two states).

### Consistency among raters

For the nine plans evaluated, federal staff assigned slightly higher average ratings than state staff (federal mean of nine states = 3.5 vs 3.2 for state staff). The paid independent public health expert's average score fell between federal and state scores (mean = 3.3). The interclass correlation coefficient (Shrout–Fleiss) for the overall plan quality scores was 0.78.

### Plan quality and other variables

Spearman rank correlation coefficients (r^s^) were consistently low and not significant at* P *≤.05 between state plan quality and 1) prevalence of adult obesity in the state (23) (r^s^ = 0.43; *P* = .24); 2) state resident population size as of the year 2000 (24) (r^s^ = 0.30; *P* = .43); 3) personal income per capita as of the year 2000 (24) (r^s^ = −0.32; *P* = .41); 4) the number of months elapsed from initial funding of a state by the CDC Obesity Prevention Program cooperative agreement and June 1, 2003, when the evaluation commenced (r^s^ = 0.54*; P* = .13); and 5) the objective review panel score received on the state application for funding under the CDC cooperative agreement (r^s^ = 0.21, *P* = .57).

## Discussion

### Limitations

One limitation of this evaluation is that only nine state plans were assessed. Another important limitation is that, although the SPI provides a systematic and detailed format for assessing plan quality, the items require that determinations be made on the basis of the professional judgment of the rater. Because raters were not instructed on the order in which to review plans, a rater's assessment could have been influenced by the sequence in which he or she reviewed plans.

Another limitation of this evaluation is that raters in general assigned a higher overall plan quality score than the mean of their scores on the 55 items of the SPI. During telephone debriefing sessions, raters were asked whether this difference resulted from their weighting some SPI items more heavily than others. Raters indicated that they did not adjust weighting for particular items or components; rather, some raters said they were reluctant to assign a low overall score, fearing it might demoralize the state staff who wrote the plan. Therefore, the item average score rather than the overall plan quality score may provide a more unbiased assessment of plan quality.

In addition, for the five self ratings shared with the CDC, all five self-rated item average scores and overall plan quality scores were higher than the average scores assigned by outside raters. State staff had background knowledge about their own plan that was neither contained in the written plan nor available to outside raters. Even though all raters were instructed to rate only the information contained in the written plan, the background knowledge of state staff may still be reflected in the high scores on self-assessment. The discrepancy in scores may also have resulted from difficulty in objectively rating one's own work. Just as grade inflation in an academic classroom may gloss over opportunities for improvement, the tendency to raise summary scores and assign high scores during self-assessment can divert attention from aspects of a state plan that could be strengthened.

The low average component score for Resources for Implementation of Plan may not accurately reflect plan quality in this area. Although some state staff indicated that lack of information in their plan for this component did reflect a lack of development of resources for implementation, a few state staff indicated a desire to keep confidential — and secure — the resources and partnerships they had worked hard to build. They expressed reluctance to reveal details about resources to anyone outside the planning group, saying they were concerned that others might try to tap into innovative resources and thereby decrease those available for obesity efforts. For some state staff, withholding resource information from their written plan represented a strategic decision rather than a lack of planning. In contrast, state staff indicated that low scores on the component Integration of Strategies with Other Programs and Implementation of Plan reflected a true lack of fully developed plans for these activities.

To accommodate state needs and preferences for the way in which information is shared, future evaluations might assess relevant background materials as well as the state's written plan. Importantly, both state and federal staff who participated in the evaluation agreed that all SPI items should remain, especially if the SPI is used for self-ratings or to guide planning. The results of this evaluation served as the basis for further dialogue between the CDC and funded states to clarify expectations regarding plan content.

All state plans have components that could be strengthened; however, the fact that at least one of the plans scored 4.0 or higher in at least one component indicates that some state plans already contain components that are "consistently strong and often close to ideal," according to the scoring rubric of the SPI. This result is especially noteworthy since state staff wrote their plans as the SPI was being developed and did not have the benefit of the recommendations for each of the SPI components.

### Use of State Plan Index to support program improvement

From November 2003 to April 2004, states were provided with summaries of the ratings of their plan and an anonymous compilation of comments from raters. Technical assistance was provided by CDC project officers, who discussed SPI results with state staff on routine telephone calls or site visits. As a result of this evaluation, some CDC project officers informally reported to the authors that the results of this evaluation helped them by identifying plan components rated as near ideal that could be recommended as resources to states engaged in writing or revising a plan.

### Process use of evaluation

Evaluation expert Michael Q. Patton asserts that people often benefit more from skills learned by virtue of their participation in an evaluation process than from the results of an evaluation (25). Patton calls this "process use" of evaluation and notes its potential for organizational learning and development. Although this evaluation was intended to systematically assess the quality of state plans, state staff have reported benefits from process use of this evaluation. State staff who evaluated plans from other states and self-rated their own plans indicated that they gained useful insights into sound planning practices. In several instances, state staff reported that the ideal standards in the SPI helped them clarify their own expectations on the content, quality, format, and public health approach to be used in the planning process and to better translate this to their partners and stakeholders. State staff also reported that they have used the SPI as a final checklist before publishing their plan.

### Other benefits of using the State Plan Index

Throughout the process of developing the State Plan Index and conducting this evaluation, information was shared with state staff, not only in states funded by the CDC for obesity but also with nonfunded states. One state that did not receive CDC funding for planning independently conducted a self-assessment of its plan using the SPI and later shared some of the results with the CDC. In this state, all members of the state obesity planning task force reviewed the state's draft plan using the SPI as a guide. Based on this review, the task force planned specific actions they would take to address potential weaknesses: for example, adding faith-based organizations and consumers as stakeholders, restating plan objectives in measurable and time-based terms, and identifying specific ways to integrate obesity efforts with other chronic disease areas as well as across systems and agencies.

Several state staff have requested copies of the SPI used for this evaluation, indicating a desire to conduct a similar evaluation of the plans of local entities. In another state not funded for obesity programs by the CDC, staff are adapting the SPI for use in state diabetes planning efforts and intend to conduct an evaluation similar to that reported here.

Finally, the results of this evaluation have been used as a training tool. In reviewing plans that were rated high, medium, and low, new staff quickly honed their understanding of the elements of a quality plan. The use of the evaluation results as a basis for focused technical assistance and evidence-based planning of future training for state staff is a program improvement already underway at the federal level.

### Plan quality and broader public health issues

Although the evaluation reported here focuses on the CDC's Obesity Prevention Program, its results can also be related to broader public health issues. For example, although the SPI provides a comprehensive list of plan attributes that public health experts and scholars identified as ideal, future evaluations can be designed to pinpoint any components that appear particularly critical to achieving desired public health outcomes. From there, future planning processes might be streamlined or focused on key components. Even more broadly, understanding the relationship between plan quality and health outcomes also contributes to a better understanding of the return on investment for public health planning efforts. Additional long-term evaluation studies could address the following:

- Does the quality of a plan affect its utility? For example, do states with better plans use them to leverage resources more effectively?
- How can planning processes be streamlined?
- Do better state plans lead to better health outcomes?

The process of engaging stakeholders, examining data, identifying and choosing interventions, building partnerships for implementation, and organizing the writing of a plan is time-consuming and complex. This evaluation showed that when compared with a set of ideal standards, the quality of state plans was variable, and some components of every state plan examined could be strengthened. As a result of this evaluation and the feedback and comments received from outside raters, several states reported informally to the CDC that they will refine and strengthen their plans, especially when plans undergo periodic updating. In general, areas where attention should be focused to strengthen existing plans were identified as resource planning, evaluation, and integrating intervention strategies across related chronic disease programs.

The participation by state staff in this evaluation demonstrates a successful evaluation partnership and a willingness of state staff to engage in program evaluation with other states as well as with the CDC. The peer ratings of plans added credibility to the process. Moreover, the time invested in reading and rating the plans of other states and applying the SPI offered an opportunity for staff to hone their planning expertise and to become more familiar with the SPI instrument and its inherent recommendations.

Perhaps most useful to public health practice is that evaluation conducted early in the life of a program can be rapidly translated into concrete program improvements with the potential to strengthen public health efforts. The agility and ease with which state staff have adapted the CDC's evaluation process and evaluation tools to guide their new planning efforts demonstrates the resourcefulness of state and local public health professionals and their genuine commitment to quality and effectiveness.

## Figures and Tables

**Table 1:** Comparison of State Plan Index (SPI)^a^ Scores For Obesity Plans for Nine U.S. States^b^

	**Range**	**Mean**	**Median**
Item average score^c^	1.0–3.0	2.4	2.6
Average overall plan quality score^d^	2.3–4.3	3.4	3.5
Self score^e ^(5 states, rater mean = 2.6)	4.0–5.0	4.7	5.0

a The SPI is available in this issue of Preventing Chronic Disease (22). SPI ratings are assigned on a Likert scale from 0 to 5 points.

b Colorado, Florida, Massachusetts, Michigan, North Carolina, Oregon, Pennsylvania, Texas, and Washington.

c Item average score is the mean of raters’ scores for the 55 individual items in the SPI, averaged for each state.

d Overall plan quality score is a single numeric rating that represents a rater’s overall evaluation of a state plan.

e Self score is a single numeric rating made by state staff of their own plan. The self score is the overall evaluation of a plan and corresponds to overall plan quality score assigned by other raters.

**Table 2:** Evaluation of Nine State Obesity Plans^a^ Using the State Plan Index (SPI)^b^

**State Plan Index Components**		**Range**	**Mean**	**Median**
A.	Involvement of Stakeholders	1.0–4.3	2.8	2.9
B.	Presentation of Data on Disease Burden and Existing Efforts to Control Obesity	0–4.5	3.3	3.6
C.	Goals	2.5–3.7	3.0	3.0
D.	Objectives	2.4–4.2	2.9	2.8
E.	Selecting Population(s) and Strategies for Intervention	0.1–3.1	2.1	2.2
F.	Integration of Strategies with Other Programs and Implementation of Plan	0.7–2.6	1.7	1.6
G.	Resources for Implementation of Plan	0–1.6	0.7	0.5
H.	Evaluation	0.6–3.6	2.0	2.0
I.	Accessibility of Plan	0.8–4.5	2.9	3.6

a Colorado, Florida, Massachusetts, Michigan, North Carolina, Oregon, Pennsylvania, Texas, and Washington.

b The SPI is available in this issue of Preventing Chronic Disease (22). SPI ratings are assigned on a Likert scale from 0 to 5 points.
